# An analysis of 97 previously diagnosed de novo adult acute erythroid leukemia patients following the 2016 revision to World Health Organization classification

**DOI:** 10.1186/s12885-017-3528-6

**Published:** 2017-08-09

**Authors:** Shaowei Qiu, Erlie Jiang, Hui Wei, Dong Lin, Guangji Zhang, Shuning Wei, Chunlin Zhou, Kaiqi Liu, Ying Wang, Bingcheng Liu, Yuntao Liu, Benfa Gong, Xiaoyuan Gong, Sizhou Feng, Yingchang Mi, Mingzhe Han, Jianxiang Wang

**Affiliations:** 1 Department of Leukemia Therapy, Institute of Hematology and Blood Diseases Hospital, Chinese Academy of Medical Science & Peking Union Medical College (CAMS & PUMC), 288 Nanjing Road, Tianjin, 300020 People’s Republic of China; 2 Department of Stem Cell Transplantation, Institute of Hematology and Blood Diseases Hospital, Chinese Academy of Medical Science & Peking Union Medical College, Tianjin, China

**Keywords:** Acute myeloid leukemia, Acute erythroid leukemia, Myelodyspalstic syndrome, Cytogenetics

## Abstract

**Background:**

The incidence of acute erythroid leukemia subtype (AEL) is rare, accounting for 5% of cases of acute myeloid leukemia (AML), and the outcome is dismal. However, in 2016 revision to the WHO classification, the subcategory of AEL has been removed. Myeloblasts are redefined as the percentage of total marrow cells, not non-erythroid cells. Therefore, the previously diagnosed AEL cases are currently diagnosed as AML or myelodyspalstic syndrome (MDS) according to new criteria.

**Methods:**

We respectively reviewed cases of 97 de novo previously diagnosed AEL and all the patients were diagnosed as AML or MDS according to the new classification scheme, and then the clinical characteristics of these two subtypes were compared. Statistical analyses were performed by SPSS software version 18.0.

**Results:**

The median age was 37 years-old, the two-thirds of previous AEL cases were diagnosed as MDS, and there was no obvious difference between two subtypes except for male/female ratio and age. Cytogenetic, rather than MDS/AML subtypes, can better represent the prognostic factor of previously diagnosed AEL patients. When the cytogenetic risk of patients belonged to MRC intermediate category and age were below 40 years-old in previous AEL cases, the patients who received induction chemotherapy without transplantation had a similar survival compared with the patients who underwent transplantation (3-year OS: 67.2% vs 68.5%).

**Conclusions:**

Cytogenetic, rather than MDS/AML subtypes, can better represent the prognostic factor of previously diagnosed AEL patients. Transplantation was a better choice for those whose cytogenetic category was unfavorable.

**Electronic supplementary material:**

The online version of this article (doi:10.1186/s12885-017-3528-6) contains supplementary material, which is available to authorized users.

## Background

The incidence of acute erythroid leukemia subtype (AEL) is rare, accounting for 5% of cases of acute myeloid leukemia (AML), characterized by a predominant erythroid population. AEL is generally associated with an aggressive clinical course. The outcome is usually dismal and transplantation will be recommended once complete remission (CR) has been achieved. The definition of AEL has undergone several revisions, including FAB (1976, 1985) and WHO (2001) edition [1, 2].

According to WHO 2008 edition, it is ruled as a case with ≥50% bone marrow (BM) erythroidprecursors and ≥ 20%myeloblasts in the non-erythroid cells (NEC). But the cases with 20% or more blasts and dysplasia involving at least 50% of cells in 2 or more lineages or myelodyspalstic syndrome (MDS) history are moved to the category of AML with MDS-related changes, even in the face of erythroid predominance [3, 4].

However, in the 2016 revision to the WHO classification, the subtype of AEL has been cancelled [5]. Currently, myeloblasts are defined as the percentage of total marrow cells, not NEC. Therefore, the previous AEL cases are currently separated into AML or MDS according to new myeloblasts criteria. Those patients with the percentage of erythroid cells exceeded 50% and total myeloblasts exceeded 20% are diagnosed as AML, NOS. And those patients with the percentage of erythroid cells exceeded 50% and total myeloblasts were less than 20% are now diagnosed as MDS.

The main reason for the change is that AEL is similar with MDS, including morphologic characteristics, genetic profiles and prognosis by some groups [6–10]. But the above clinical studies were predominantly focused on the relationship among AEL, MDS with erythroid hyperplasia and the clinical significance of new myeloblasts criterion has not been evaluated yet. It was emphasized that the risk of cytogenetic could represent the most important prognostic factor better than myeloblasts percentage [7, 9, 10]. With the aim to clarify the clinical significance of new classification, we respectively reviewed cases of 97 de novo diagnosed AEL according to 2008WHO classification, and now all the patients were diagnosed as AML or MDS according to new classification and they were compared in the multiple aspects of the clinical characteristics, cytogenetic and molecular genetics profiles. In addition, the relationship between treatment modalities and clinical outcome was investigated among the patients.

## Methods

### Patients

All patients were diagnosed de novo acute erythroid/myeloid leukemia, while pure erythroid leukemia, AML with MDS-related changes and recurrent genetic abnormalities were precluded. All study regimens were in accordance with the Declaration of Helsinki and approved by the institutional review boards of the hospital. All patients were investigated by morphology, bone marrow aspiration and cytogenetic analysis. They all provided written informed consent for sample analyses. All the patients received DA (daunorubicin 40-45 mg/m^2^ on day1–3 and cytarabine 100-150 mg/m^2^ on day 1–7) or HAA (Homoharringtonine 2 mg/m^2^ on day 1–7,cytarabine 100-150 mg/m^2^ on day 1–7 and aclarubincin 20 mg/d on day 1–7) or HAD (Homoharringtonine 2 mg/m^2^ on day 1–7,cytarabine 100-150 mg/m^2^ on day 1–7 and daunorubicin 40 mg/m^2^ on day 1–3) as induction regimens. Once CR had been achieved, all the patients were suggested to receive allogeneic hematopoietic stem cell transplantation. If the patients cannot afford transplantation or cannot find suitable donor, they would receive HD-Ara-C (cytarabine 3 g/m^2^ per 12 h on day1–3) or MA (mitoxantrone 8 mg/m^2^ on day 1–3 and cytarabine 1.5 g/m^2^ per 12 h on day1–3) or DA (daunorubicin 40 mg/m^2^ on day 1–3 and cytarabine 1.5 g/m^2^ per 12 h on day1–3) as consolidation regimens. Clinical follow-up information, including overall survival (OS) and disease free survival (DFS) were retrieved from the electronic medical record and telephone communication. The median follow up was 21.9 month, the last follow-up time was 31/8/2016.

### Conventional cytogenetics

At least twenty metaphases were analyzed in chromosomal analysis. The results were recorded adopting the International system for Human Cytogenetic Nomenclature.

### Molecular mutation analysis

Gene mutation analysis was performed for *FLT3-ITD* (*n* = 69), *NPM*1 (*n* = 47), *CEBPA-BZIP* (*n* = 47), *CEBPA-TAD* (*n* = 47), *C-kit exon8* (*n* = 47), *C-kit exon17* (*n* = 47), *DNMT3A* (*n* = 33), *IDH1* (*n* = 16), *IDH2* (*n* = 16).

### Statistical analyses

The Fisher exact test and χ^2^test were applied to variables. OS was defined as the time from diagnosis to death or last follow-up time. DFS was defined as the time from complete remission (CR) to relapse or death or last follow-up time. OS and DFS were estimated by Kaplan-Meier method. Survival curves were compared by the log-rank test. Multivariate analysis was performed by Cox proportional regression model. All tests were two-sided, accepting *p* value of 0.05 or below as indicating a statistically significant difference. Statistical analyses were performed by SPSS software version 18.0.

## Results

### Clinical characteristics, cytogenetic analysis and molecular mutations in MDS and AML subtypes

Total 97 patients were previously diagnosed as de novo AEL following WHO2008 criteria from 2004 to 2016. According to the new criteria, of them 65 patients were modified as MDS, 32 patients were diagnosed as AML, NOS. Therefore, majority of previous AEL were diagnosed as MDS according to the new classification criterion. The clinical features of total cases were summarized in Table 1. As shown, incidence was higher in male in totally, particular MDS cases. The median age of total cases was 37 years old. And the age of MDS cases was older than that of AML cases (39 vs 33, *p* = 0.044). However, the median hemoglobin, white blood cell count, platelet count was nearly the same in both cases.

**Table 1:** Characteristics of 97 previous diagnosed de novo acute erythroid/myeloid leukemia patients

	Total *N* = 97	MDS *N* = 65	AML *N* = 32	*P*
Characteristics				
Sex, M/F	60/37	46/19	14/18	0.01
Median age, y(range)	37 (11–75)	39 (11–70)	33 (15–75)	0.044
Median hemoglobin level, g/L(range)	72 (36–141)	71 (36–141)	73 (41–119)	0.615
Median WBC, ×10^9^/L(range)	3.5 (0.61–62.9)	3.36 (0.61–44.83)	4.25 (1.21–62.9)	0.201
Median platelet count, ×10^9^/L(range)	49 (8–480)	49 (9–480)	46 (8–233)	0.441
Cytogenetic results, %				
Normal karyotype	77.8 (70/90)	76.3 (45/59)	80.6 (25/31)	0.635
Complex karyotype	10 (9/90)	11.9 (7/59)	6.5 (2/31)	0.657
Monosomy karyotype	5.6 (5/90)	5.1 (3/59)	6.5 (2/31)	1.0
Intermediate MRC category	87.8 (79/90)	86.4 (51/59)	87.5 (28/32)	1.0
Unfavorable MRC category	12.2 (11/90)	13.6 (8/59)	9.4 (3/32)	0.804
Good IPSS category	78.9 (71/90)	78.0 (46/59)	80.6 (25/31)	0.767
Intermediate IPSS category	10 (9/90)	10.2 (6/59)	9.7 (3/31)	1.0
Adverse IPSS category	11.1 (10/90)	11.9 (7/59)	9.7 (3/31)	1.0
Molecular mutations, %				
*NPM1* mutation	19.1 (9/47)	18.2 (6/33)	21.4 (3/14)	1.0
*FLT3-ITD* mutation	4.3 (3/69)	6.5 (3/46)	4.3 (1/23)	1.0
*CEBPA* single mutation	6.4 (3/47)	6.3 (2/32)	6.7 (1/15)	0.487
*CEBPA* double mutation	6.4 (3/47)	3.1 (1/32)	13.3 (2/15)	0.487
*DNMT3A* R882 mutation	9.1 (3/33)	11.5 (3/26)	0 (0/7)	1.0

Further, the chromosome karyotype were investigated. The results were available for 90 patients, including 59 MDS cases and 31 AML cases. Totally, the proportion of aberrant karyotype accounted for 20%, there were no difference in the proportion of normal karyotype, complex karyotype and monosomy karyotype between two subtypes. The proportions of each cytogenetic risk category using the IPSS and UKMRC schemes were also similar in both cases. Following MRC category, the majority of patients belonged to intermediate risk (87.8%), only 12.2% patients belonged to unfavorable risk.

Finally, some specific molecular mutations were further investigated. Only 69 cases were assessed for *FLT3-ITD* mutation, 47 cases were assessed for *NPM1* mutation, *CEBPA* mutation (*B-ZIP* domain and *TAD* domain), *C-kit* mutation, 33 cases were assessed for *DNMT3A* mutation. The incidence of the above mutations was 19.1% (*NPM1*), 4.3% (*FLT3-ITD*), 6.4% (*CEBPA single*), 6.4% (*CEBPA* double) and 9.1% (*DNMT3A*)separately. However, there were no difference in the incidence of these mutations in both cases. In addition, *C-kit* mutations were not found in these patients.

### Survival according to MDS/AML subtypes and cytogenetic risk category

**Fig. 1:**
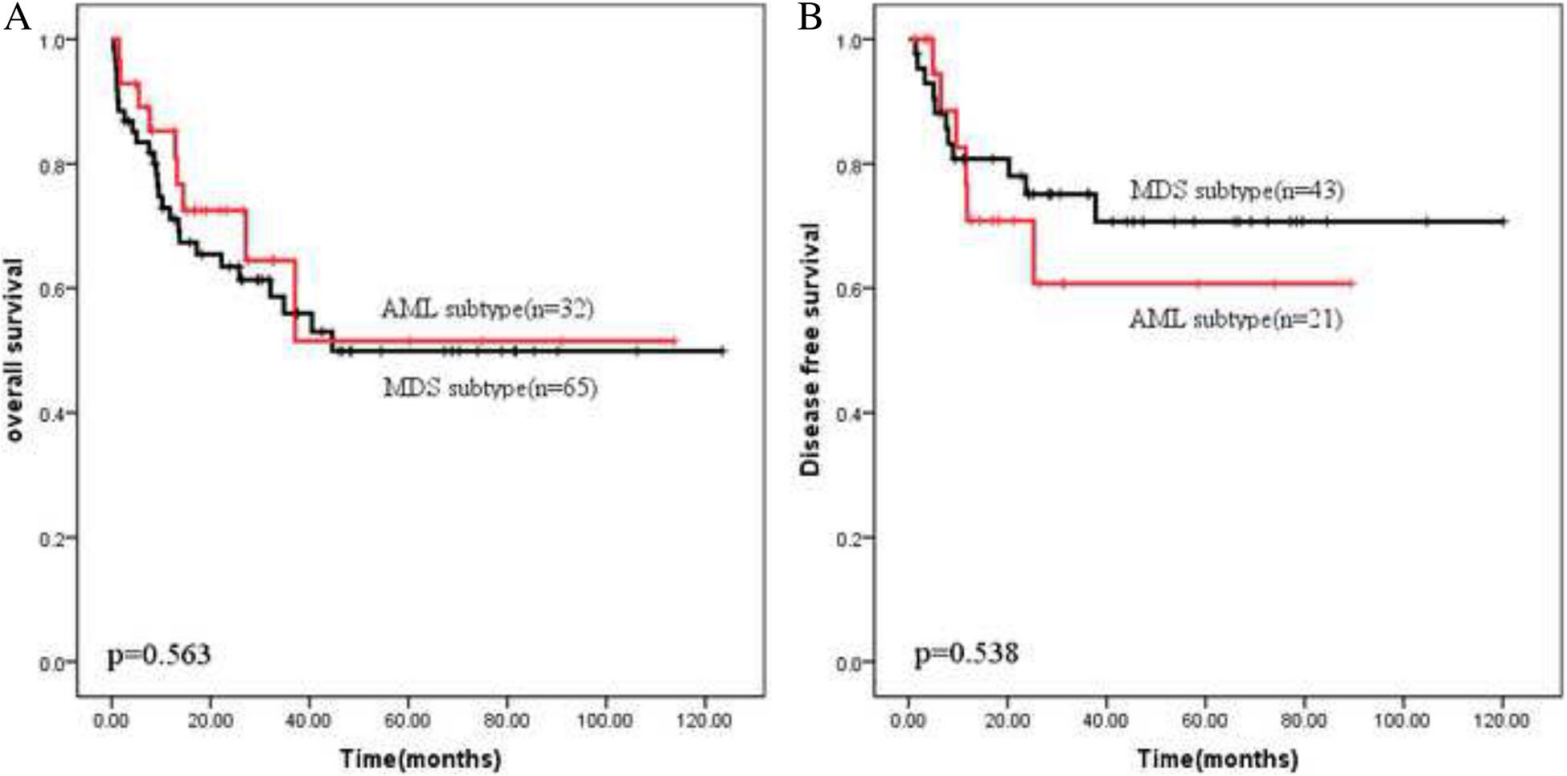
Overall survival (**a**) and disease free survival (**b**) depending on MDS vs AML subtype

Survival of the total AEL patients was firstly investigated by MDS vs AML subtype, as presented in Fig. 1 and Table 2, the 3-year OS was 56% (MDS subtype) and 64.4% (AML subtype) respectively. The median OS of MDS subtype was 44.6 months. And median OS of AML subtype had not been reached. The 3-year DFS was 75.1% (MDS subtype) and 60.7% (AML subtype) respectively. There were no difference between these two subtype in OS and DFS (*p* > 0.05).

**Table 2:** Median 3-year overall survival (OS)/disease free survival (DFS) and median OS/DFS in months in previously diagnosed AEL patients depending on subtypes and cytogenetic risk group (MRC and IPSS criteria)

Parameter	Median 3-year OS	Median OS (Months)	*P*	Median 3-year DFS	Median DFS (Months)	*P*
Subtype						
MDS	56%	44.6	0.563	75.1%	n.r.	0.538
AML	64.4%	n.r.		60.7%	n.r.	
Cytogenetics						
Intermediate MRC category	62.2%	n.r	<0.0001	71.5%	n.r	0.008
Unfavorable MRC category	12%	5.03		25%	5.3	
Good IPSS category	56.9%	n.r	0.045	69.3%	n.r	0.099
Intermediate IPSS category	75%	n.r		85.7%	n.r	
Adverse IPSS category	13.3%	11.8		25%	5.33	

*Abbreviation*: *IPSS* International Prognostic Scoring System, *MRC* Medical Research Council

**Fig. 2:**
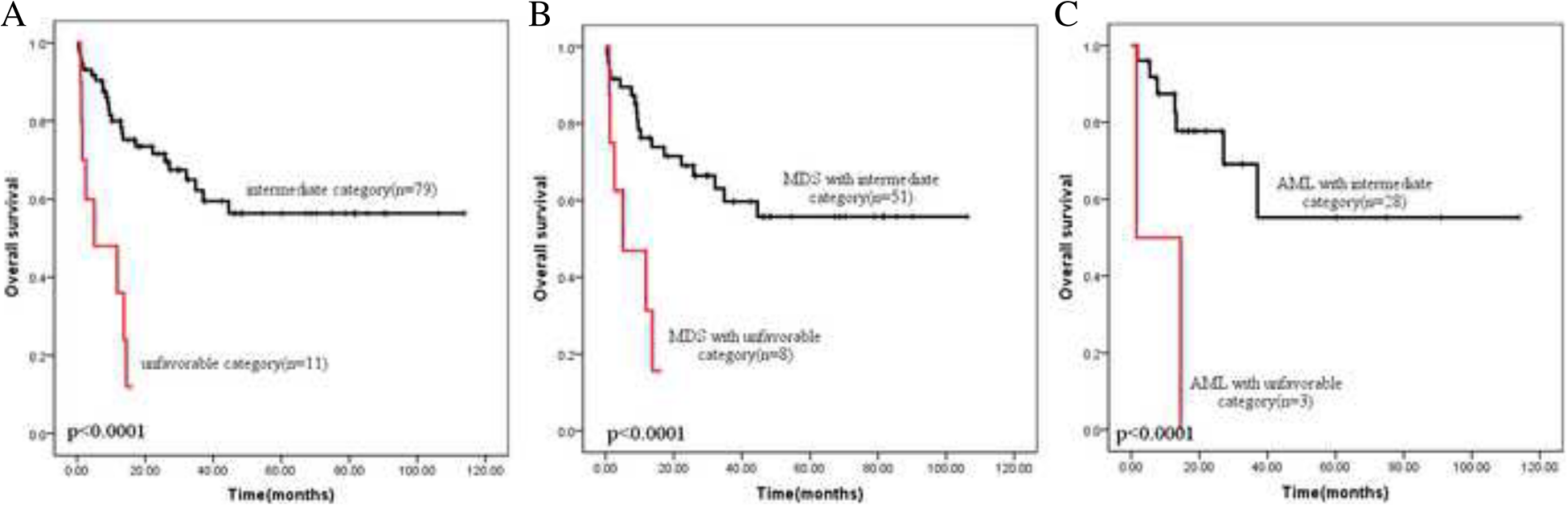
Overall survival depending on the cytogenetic category group (MRC criteria). Unfavorable category was associated with the significantly worse outcome compared with intermediate category. **a** in all previous diagnosed AEL patients (*n* = 90, *p* < 0.0001); **b** in the current MDS subtypes (*n* = 59, *p* < 0.0001); **c** in the current AML subtypes (*n* = 31, *p* < 0.0001)

**Fig. 3:**
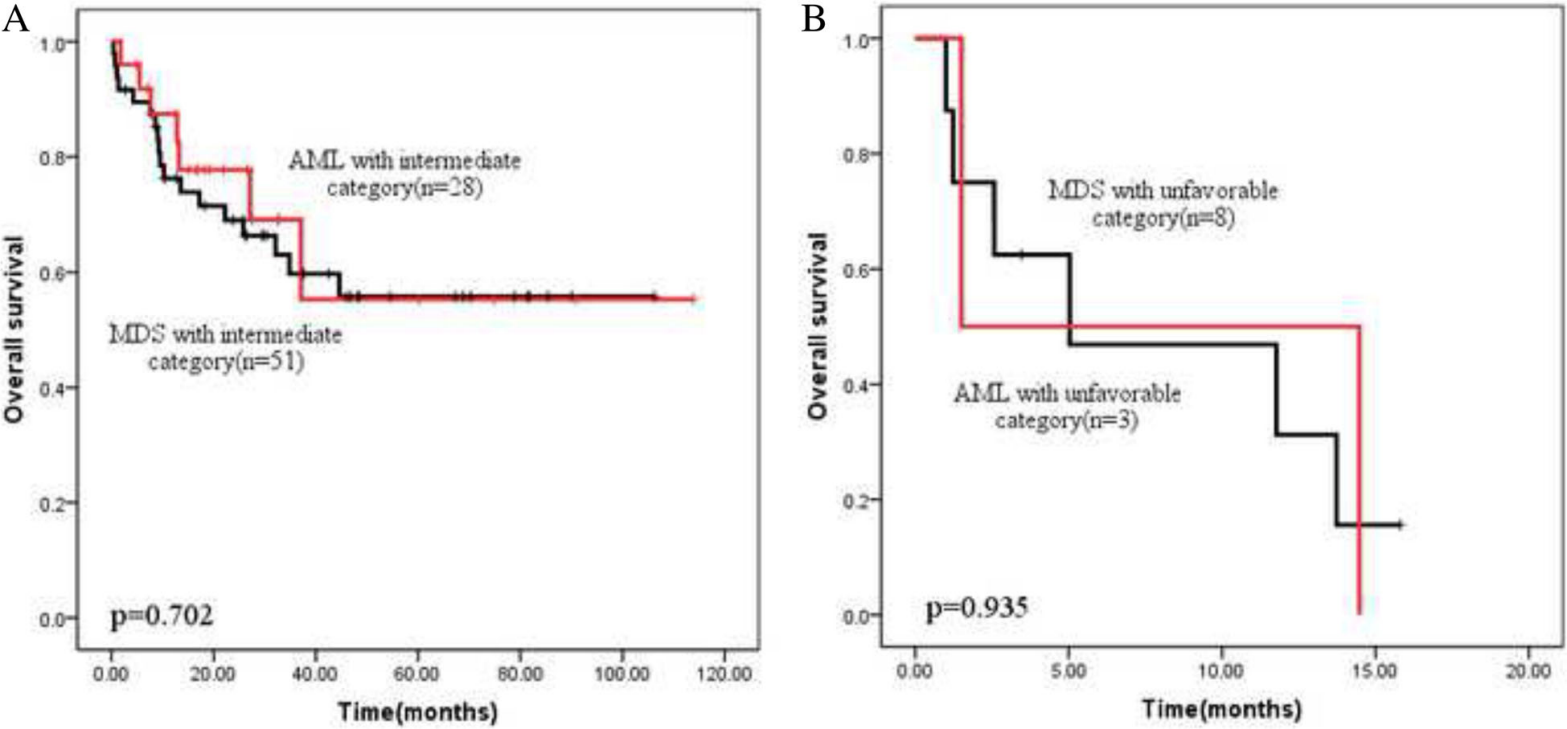
Overall survival depending on the cytogenetic risk group (MRC criteria) in current MDS and AML subtypes. **a** in the intermediate category (*n* = 79); **b** in the unfavorable category (*n* = 11)

Further, the survival was explored according to cytogenetic category in all 97 patients. The MRC criteria and IPSS category were both investigated, as Table 2 showed, MRC category might be more suitable for distinguishing clinical outcome than IPSS category. As shown in the Fig. 2a, unfavorable karyotype was associated with an observable inferior 3-year OS compared to intermediate karyotype (12% vs 62.5%, *p* = 0.000), the results occurred both in MDS subtype and AML subtype (Fig. 2b and c). However, the survival of same category in MDS subtype or AML subtype did not show any difference (Fig. 3). The 3-year OS of intermediate category in MDS and AML subtype was similar (59.7% vs 69.1%, *p* = 0.706), so were the median OS of unfavorable category (MDS 5.03 month vs AML 1.5 month, *p* > 0.05).

### The prognostic factors in AEL

When we analyzed various clinical characteristics, including MDS vs AML subtype, cytogenetic and molecular mutations with regards to their ability to predict overall survival of all 97 patients (Table 3), only cytogenetic risk and age were related. As shown in Additional file 1: Fig. S1, with the age increased, the survival decreased significantly. The 3-year OS of age < 40, 40–60, >60 was 65%, 52.3% and 33.3% respectively (*p* = 0.013). As shown in Additional file 2: Table S1, with the age increased, the proportion of unfavorable cytogenetic category increased sharply, the ratio was only 7.8% in age < 40 group, but when age was older than 60, the ratio was 25%. However, MDS vs AML subtype, gender, WBC, *NPM1* mutation, *FLT3-ITD* mutation have little impact on the survival. Further, adopting multivariate analysis, the cytogenetic classification (*p* = 0.000, RR = 5.614) became the only independent parameter that implied outcome as shown in Table 3, the results confirmed that cytogenetic, rather than arbitrary myeloblast percentage of total marrow cells, can better represent the outcome of patients.

**Table 3:** Analysis of prognostic parameters regarding overall survival in univariate and multivariate analyses in the 97 previous diagnosed AEL patients

Variable	Univariate analysis	Multivariate analysis			
	*P*	*P*	HR	95% CI for HR	
				Upper	Lower
Age(20 years increase)	0.013	0.1	1.546	2.600	0.920
Gender	0.436	0.462	0.609	2.281	0.163
White blood count	0.75	0.908	0.961	1.901	0.486
Platelet count	0.457	0.608	1.551	8.309	0.289
Hemoglobin level	0.092	0.271	3.256	26.624	0.398
MDS verse AML	0.563	0.916	1.059	3.084	0.364
Cytogenetic risk group(MRC)	<0.0001	<0.001	5.614	13.137	2.399
*NPM1* mutation	0.058	0.154	0.223	1.757	0.028
*FLT3-ITD* mutation	0.593	0.736	1.461	13.189	0.162

*HR* hazard ratios, *CI* confidence intervals

### The effect of transplantation in AEL

The influence of the treatment modalities was also investigated. Of total 97 patients, 70 patients received chemotherapy or best supportive treatment as their treatment choice. 27 patients received transplantation eventually after chemotherapy, of them 25 patients attained complete remission (CR) before transplantation. The distribution of treatment choice in clinical characteristics such as cytogenetic category, age group and MDS vs AML subtype were listed in Table 4. In the intermediate cytogenetic risk, 31.6% of patients received transplantation. The proportion of transplantation patients in the age < 40 and 40–60 group was 40.7% and 14.7%. The proportion of transplantation cases in AML or MDS subtype patients was similar, 25% (AML) vs 29.2% (MDS).

**Table 4:** The proportion of treatment modalities (no transplantation vs transplantation) in different cytogenetic category, age group and disease subtypes

		no transplantation	transplantation
Cytogenetic category	Intermediate, *n* = 79	68.4% (54/79)	31.6% (25/79)
	Unfavorable, *n* = 11	100% (11/11)	0 (0/11)
Age group	<40, *n* = 54	59.3% (32/54)	40.7% (22/54)
	40–60, *n* = 34	85.3% (29/34)	14.7% (5/34)
	>60, *n* = 9	100% (9/9)	0 (0/9)
Subtypes	MDS, *n* = 65	70.8% (46/65)	29.2% (19/65)
	AML, *n* = 32	75% (24/32)	25% (8/32)

**Fig. 4:**
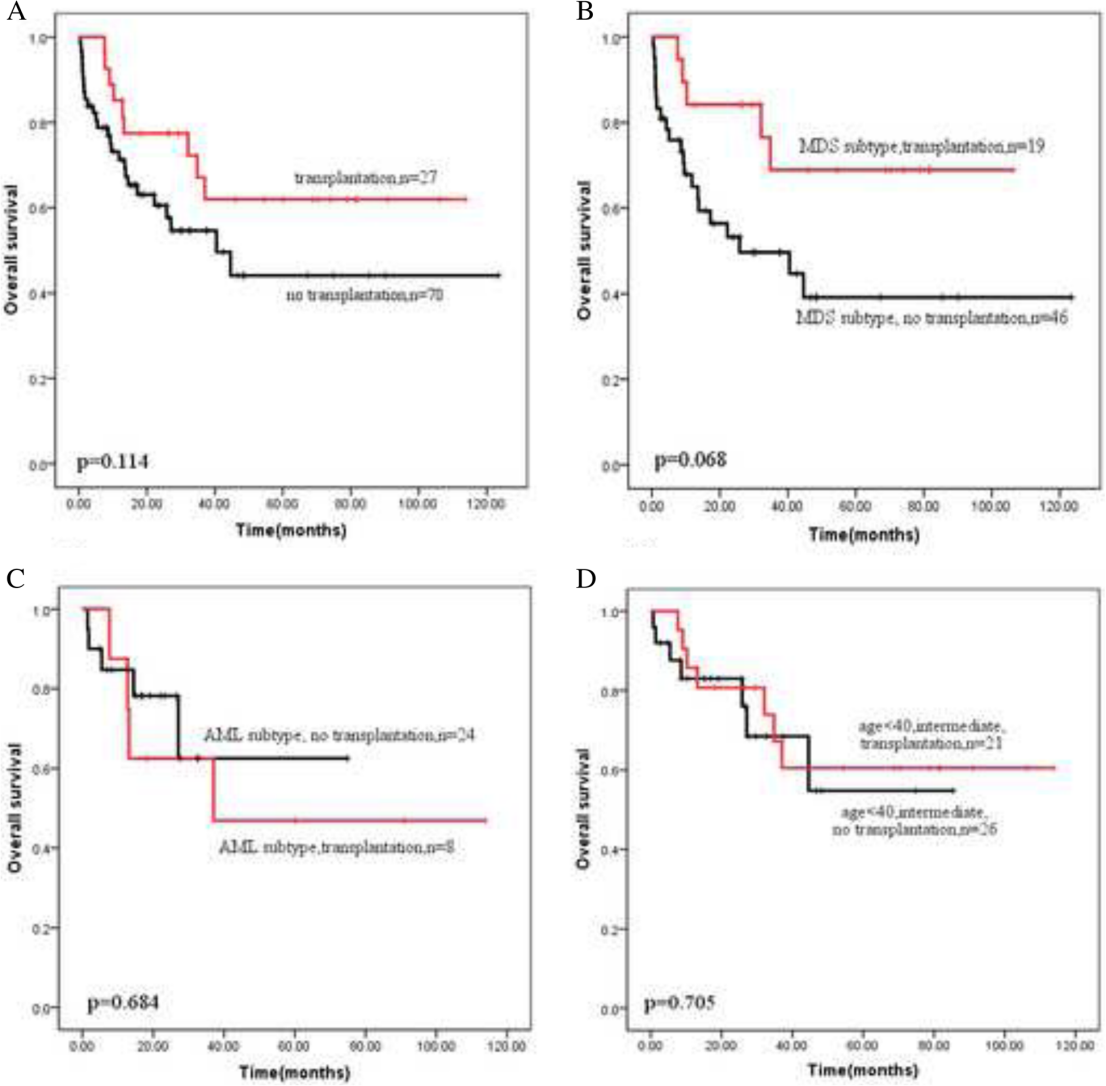
Overall survival depending on treatment modalities (transplantation vs no transplantation). **a** in the previous diagnosed 97 AEL patients (*p* = 0.114); **b** in the current 67 MDS patients (*p* = 0.068); **c** in the current AML patients (*p* = 0.684); **d** in those whose cytogenetic risk belonged to intermediate category and age were below 40 years-old (*p* = 0.705)

The survival of transplantation cases and chemotherapy/supportive treatment cases were shown in Fig. 4. Totally, the 3-year OS of the two groups were 67.1% and 54.6%, there was no significant difference (*p* = 0.114) (Fig. 4a). The survival of transplantation was further explored in MDS and AML subtype. In MDS subtype, the 3-year OS of two groups was 68.9% and 49.7% respectively (*p* = 0.068) (Fig. 4b). However, in AML subtype, the difference of 3-year OS was similar (62.5% vs 62.6%, *p* > 0.05) (Fig. 4c). Further analysis showed that when the cytogenetic risk of patients belonged to MRC intermediate risk and age were below 40 years-old, the patients who received chemotherapy without transplantation had a similar survival compared with the patients who underwent transplantation (3-year OS: 67.2% vs 68.5%) as Fig. 4d shown.

## Discussion

In our study, previously diagnosed AEL cases were modified as AML subtype or MDS subtype following the new classification. The majority of AEL cases were diagnosed as MDS, accounting for 2/3 of the patients. After comparing the clinical characteristics, cytogenetic risk and molecular mutation of two subtypes, we found that there were no difference between two subtypes except for male/female ratio and age. When the survival of previously AEL cases was analyzed, only MRC cytogenetic category and age were correlated with survival, regardless of MDS/AML subtype. Transplantation was beneficial for all the patients, particular MDS patients. However, when the patients whose age were below 40 and cytogenetic risk belonged to the intermediate category, the survival of those who only receive chemotherapy was similar to that of the patients who received transplantation eventually. Therefore, taking expensive cost and severe complication of transplantation into consideration, it was suggested this population of patients could receive chemotherapy as main treatment choice.

In contrast to Western studies, the most noticeable point of our study was that the median age of our patients was 37-years old, much younger than the data of Western studies [11, 12]. Owing to younger age and less unfavorable cytogenetic, the 3-year OS of our patients could reach 50–60%, better than the survival of published articles of AEL.

As we known, there is no specific chromosome abnormality described in AEL. Complex karyotypes with multiple structure abnormality are common, −5/del(5q), −7/del(7q) and +8 were the most common subtypes [13]. The impact of cytogenetic on survival of AEL had been confirmed by other colleagues [7, 9]. The conclusion was further confirmed by our study in the application of previous diagnosed AEL patients. In addition, MRC category was better than IPSS category for distinguishing the outcomes, but that was against by Liu CJ et al. [14] IPSS category was not suitable for the distinction between good and intermediate category.

There were also some disadvantages in the study. The samples for the assessment of molecular mutations were not huge enough. Few cases had been assessed at the molecular level owing to the rarity of AEL in Western countries. Recently, the impact of molecular mutations on the survival has been intrigued more and more attentions. It was believed that AEL could be distinguished by cytogenetic and molecular genetic characteristics, such as *RUNX1* and *TP53* mutations that implied the worse outcome and *NPM1* mutation that implied better outcome [7]. But in our study, *FLT3-ITD*, *NPM1*mutation did not show any significance for prognosis to date. Therefore, there was still a long time for us to accumulate the samples to clarify the issue.

To date, there were few articles investigating the application of new classification on the previous diagnosed AEL cases. Following WHO new scheme, the subtype of previous AEL was removed and substituted for AML or MDS. In our study, after comparing the two subtypes, there was no obvious difference in reality, but giving the relationship among AEL, AML –MRC with erythroid hyperplasia and MDS with erythroid hyperplasia by a serial of researches [6–10, 15], it was still reasonable to adopt new classification.

In our impression, it has a dismal outcome for AEL patients. Stem cell transplantation can substantially improve the outcome of the disease, with 5-year leukemia-free survival reaching approximately 60% after HLA-identical sibling SCT [16]. But our study suggested that when the cytogenetic risk of patients belonged to MRC intermediate category and age were below 40 years-old, the patients who received induction chemotherapy without transplantation had a similar survival compared with the patients who underwent transplantation, the 3-year OS of both patients was 60–70%. The conclusion was also supported by the researches that were conducted in MD Anderson [17]. Their data showed that the median OS of patients with AEL was 36 weeks and the diagnose of AEL did not imply a dismal outcome. Transplantation was a better choice for those whose cytogenetic category was unfavorable.

## Conclusions

Following the new classification, there were no differences between MDS and AML subtype except for male/female ratio and age. Cytogenetic, rather than MDS/AML subtypes, can better represent the prognostic factor of previously diagnosed AEL patients. Transplantation was a better choice for those whose cytogenetic category was unfavorable.

## Additional files

Additional file 1: Figure S1. The 3-year OS of 97 previously diagnosed de novo adult AEL patients according to age group. The 3-year OS of <40, 40–60 and >60 age group. (TIFF 170 kb)

Additional file 2: Table S1. The ratio of different cytogenetic risk category in different age group. The ratio of different cytogenetic risk category (intermediate and unfavorable risk) in <40, 40–60, >60 age group. (DOCX 12 kb)

Additional file 3: Raw data. The clinical characteristic of 97 previously diagnosed de novo adult acute erythroid leukemia patients. The clinical characteristic of 97 previously diagnosed de novo adult acute erythroid leukemia patients were listed, including MDS/AML subtype, MRC cytogenetic risk, survival data, gene mutation and so on. (DOC 239 kb)

## Abbreviations

AEL: Acute erythroid leukemia subtype
AML: Acute myeloid leukemia
CR: Complete remission
DFS: Disease free survival
IPSS: International Prognostic Scoring System
MDS: Myelodyspalstic syndrome
MRC: Medical Research Council
NOS: Not otherwise specified
OS: Overall survival
WHO: World Health Organization
