# Supplementary figures and images for: An analysis of 97 previously diagnosed de novo adult acute erythroid leukemia patients following the 2016 revision to World Health Organization classification
Source: BMC Cancer. 2017 Aug 9;17:534. doi: 10.1186/s12885-017-3528-6 (PMC5550989; doi:10.1186/s12885-017-3528-6)

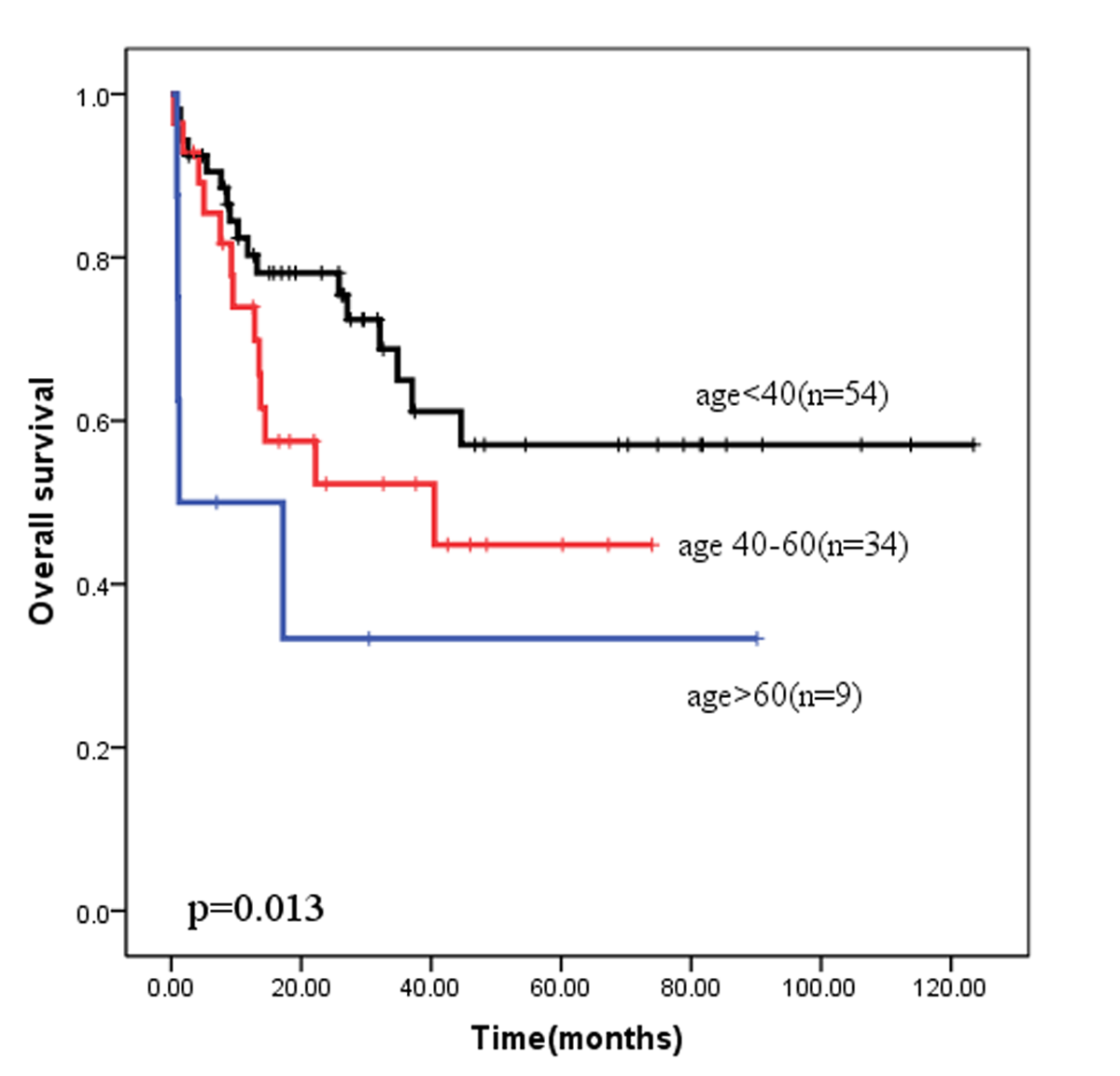

Supplement: Supplementary file 1 — The 3-year OS of 97 previously diagnosed de novo adult AEL patients according to age group. The 3-year OS of <40, 40–60 and >60 age group. (TIFF 170 kb) [file 12885_2017_3528_MOESM1_ESM.tif]
