# Supplementary material for: An analysis of 97 previously diagnosed de novo adult acute erythroid leukemia patients following the 2016 revision to World Health Organization classification
Source: BMC Cancer. 2017 Aug 9;17:534. doi: 10.1186/s12885-017-3528-6 (PMC5550989; doi:10.1186/s12885-017-3528-6)
Supplement: Supplementary file 2 — The ratio of different cytogenetic risk category in different age group. The ratio of different cytogenetic risk category (intermediate and unfavorable risk) in <40, 40–60, >60 age group. (DOCX 12 kb) [file 12885_2017_3528_MOESM2_ESM.docx]

Table S1 The ratio of different cytogenetic risk category in different age group.

|  | Intermediate risk | Unfavorable risk |
| --- | --- | --- |
| <40,n=51 | 92.2%(47/51) | 7.8%(4/51) |
| 40-60,n=31 | 83.9%(26/31) | 16.1%(5/31) |
| >60,n=8 | 75%(6/8) | 25%(2/8) |
