# Supplementary material for: An analysis of 97 previously diagnosed de novo adult acute erythroid leukemia patients following the 2016 revision to World Health Organization classification
Source: BMC Cancer. 2017 Aug 9;17:534. doi: 10.1186/s12885-017-3528-6 (PMC5550989; doi:10.1186/s12885-017-3528-6)
Supplement: Supplementary file 3 — Raw data. The clinical characteristic of 97 previously diagnosed de novo adult acute erythroid leukemia patients. The clinical characteristic of 97 previously diagnosed de novo adult acute erythroid leukemia patients were listed, including MDS/AML subtype, MRC cytogenetic risk, survival data, gene mutation and so on. (DOC 239 kb) [file 12885_2017_3528_MOESM3_ESM.doc]

| ID | Subtype | MRC | IPSS | OS | DFS | CR | Transplantation | Survival | Age range | WBC | Hb | PLT | ITD | NPM1 | CEBPA | DNMT3A |
| --- | --- | --- | --- | --- | --- | --- | --- | --- | --- | --- | --- | --- | --- | --- | --- | --- |
| 1 | MDS | Intermediate | Good | 22.20 |  | No | No | Death | 40-60 | 5.5 | 57 | 60 | ND | ND | ND | ND |
| 2 | AML | Intermediate | Good | 26.53 |  | No | No | Survival | <40 | 1.21 | 57 | 23 | ND | ND | ND | ND |
| 3 | MDS | Intermediate | Good | 25.80 | 23.70 | YES | No | Death | <40 | 5.2 | 69 | 71 | ND | ND | ND | ND |
| 4 | MDS | Intermediate | Intermediate | 48.20 | 10.97 | YES | No | Survival | <40 | 7.81 | 68 | 38 | ND | ND | ND | ND |
| 5 | AML | Unkown | Unkown | 23.13 | 21.27 | YES | No | Survival | <40 | 14.8 | 96 | 59 | ND | ND | ND | ND |
| 6 | MDS | Intermediate | Good | 67.27 | 65.87 | YES | No | Survival | 40-60 | 1.9 | 61 | 20 | ND | ND | ND | ND |
| 7 | MDS | Intermediate | Intermediate | 90.13 |  | No | No | Survival | >60 | 1.3 | 60 | 70 | ND | ND | ND | ND |
| 8 | MDS | Intermediate | Good | 9.27 | 7.57 | YES | No | Death | 40-60 | 5.8 | 79 | 36 | ND | ND | ND | ND |
| 9 | MDS | Unkown | Unkown | 123.47 | 120.17 | YES | No | Survival | <40 | 1.8 | 84 | 13 | ND | ND | ND | ND |
| 10 | AML | Intermediate | Good | 74.87 | 73.87 | YES | No | Survival | <40 | 43.7 | 77 | 28 | ND | ND | ND | ND |
| 11 | MDS | Intermediate | Intermediate | 85.43 | 84.50 | YES | No | Survival | <40 | 1.4 | 77 | 84 | ND | ND | ND | ND |
| 12 | MDS | Intermediate | Good | 9.03 | 8.00 | YES | YES | Death | <40 | 2.4 | 71 | 51 | ND | ND | ND | ND |
| 13 | MDS | Intermediate | Good | 1.07 |  | No | No | Death | >60 | 3.1 | 112 | 65 | ND | ND | ND | ND |
| 14 | MDS | Intermediate | Good | 32.10 | 20.27 | YES | YES | Death | <40 | 1.8 | 50 | 13 | ND | ND | ND | ND |
| 15 | AML | Intermediate | Good | 16.93 |  | No | No | Survival | <40 | 7.2 | 53 | 94 | ND | ND | ND | ND |
| 16 | AML | Intermediate | Good | 113.80 | 3.97 | YES | YES | Survival | <40 | 4.6 | 74 | 11 | ND | ND | ND | ND |
| 17 | MDS | Intermediate | Good | 7.97 | 6.43 | YES | No | Survival | 40-60 | 2.3 | 67 | 243 | ND | ND | ND | ND |
| 18 | AML | Intermediate | Good | 8.17 | 1.23 | YES | No | Survival | <40 | 1.54 | 89 | 47 | ND | ND | ND | ND |
| 19 | MDS | Intermediate | Intermediate | 106.20 | 104.70 | YES | YES | Survival | <40 | 2.95 | 101 | 84 | ND | ND | ND | ND |
| 20 | MDS | Intermediate | Good | 10.33 | 9.37 | YES | No | Survival | <40 | 44.83 | 36 | 9 | ND | ND | ND | ND |
| 21 | AML | Intermediate | Good | 7.67 | 6.47 | YES | YES | Death | <40 | 2.6 | 54 | 31 | Neg | ND | ND | ND |
| 22 | MDS | Intermediate | Good | 2.70 | 1.73 | YES | No | Survival | <40 | 5.9 | 86 | 73 | ND | ND | ND | ND |
| 23 | MDS | Unkown | Unkown | 40.50 | 37.77 | YES | No | Death | 40-60 | 10.66 | 64 | 32 | ND | ND | ND | ND |
| 24 | AML | Intermediate | Good | 90.93 | 89.43 | YES | YES | Survival | <40 | 11.9 | 99 | 41 | Neg | ND | ND | ND |
| 25 | AML | Intermediate | Good |  |  | No | No | Unkown | >60 | 31.59 | 78 | 233 | Neg | ND | ND | ND |
| 26 | AML | Intermediate | Good | 21.97 |  | No | No | Survival | 40-60 | 8.24 | 72 | 113 | Neg | ND | ND | ND |
| 27 | MDS | Intermediate | Good | 81.80 | 78.30 | YES | YES | Survival | <40 | 2.59 | 66 | 39 | Neg | ND | ND | ND |
| 28 | MDS | Intermediate | Good | 81.37 | 79.53 | YES | YES | Survival | <40 | 1.2 | 73 | 82 | Neg | ND | ND | ND |
| 29 | AML | Intermediate | Good | 19.10 | 18.17 | YES | No | Survival | <40 | 2.24 | 65 | 45 | Neg | ND | ND | ND |
| 30 | MDS | Intermediate | Good | 78.87 | 77.13 | YES | YES | Survival | <40 | 1.36 | 74 | 33 | Neg | ND | ND | ND |
| 31 | MDS | Unkown | Unkown |  |  | No | No | Unkown | 40-60 | 1.9 | 40 | 23 | Neg | ND | ND | ND |
| 32 | MDS | Unfavorable | Adverse | 2.57 |  | No | No | Death | <40 | 2.14 | 42 | 17 | ND | ND | ND | ND |
| 33 | AML | Unfavorable | Adverse |  |  | No | No | Unkown | 40-60 | 3.2 | 76 | 66 | Neg | ND | ND | ND |
| 34 | MDS | Unfavorable | Adverse | 11.77 | 1.77 | YES | No | Death | <40 | 6.5 | 46 | 21 | Neg | ND | ND | ND |
| 35 | AML | Intermediate | Good | 12.83 | 11.77 | YES | YES | Death | 40-60 | 4.8 | 60 | 28 | Neg | ND | ND | ND |
| 36 | MDS | Unkown | Unkown | 74.00 | 72.53 | YES | YES | Survival | 40-60 | 3.58 | 79 | 79 | ND | ND | ND | ND |
| 37 | MDS | Unfavorable | Adverse | 5.03 |  | No | No | Death | 40-60 | 4.1 | 67 | 43 | Neg | ND | ND | ND |
| 38 | MDS | Unfavorable | Adverse | 71.80 | 57.70 | YES | No | Survival | <40 | 6 | 74 | 24 | Neg | ND | ND | ND |
| 39 | AML | Intermediate | Intermediate | 27.10 | 4.90 | YES | No | Death | <40 | 3.85 | 80 | 8 | Pos | ND | ND | ND |
| 40 | MDS | Intermediate | Good | 70.30 | 69.17 | YES | YES | Survival | <40 | 18.9 | 105 | 35 | Neg | ND | ND | ND |
| 41 | MDS | Intermediate | Intermediate | 68.80 | 66.70 | YES | YES | Survival | <40 | 1.33 | 56 | 20 | Neg | ND | ND | ND |
| 42 | MDS | Unkown | Unkown | 0.90 |  | No | No | Death | >60 | 4.3 | 77 | 103 | Neg | Neg | Neg | ND |
| 43 | AML | Unfavorable | Adverse | 1.50 |  | No | No | Death | <40 | 62.9 | 61 | 55 | Neg | Neg | Neg | ND |
| 44 | MDS | Intermediate | Good | 9.50 |  | No | No | Death | 40-60 | 8.4 | 44 | 28 | Pos | Neg | Neg | ND |
| 45 | AML | Intermediate | Good | 1.77 |  | No | No | Death | 40-60 | 38.4 | 65 | 92 | Neg | Neg | Neg | ND |
| 46 | MDS | Unfavorable | Adverse | 3.47 |  | No | No | Survival | 40-60 | 3.7 | 82 | 46 | Neg | ND | ND | ND |
| 47 | AML | Intermediate | Intermediate | 60.20 | 58.47 | YES | YES | Survival | 40-60 | 3.9 | 76 | 72 | Neg | Neg | Neg | ND |
| 48 | MDS | Intermediate | Good | 4.23 | 1.40 | YES | No | Death | 40-60 | 1.5 | 49 | 66 | Neg | ND | ND | ND |
| 49 | AML | Intermediate | Good | 37.07 | 25.27 | YES | YES | Death | <40 | 1.9 | 55 | 27 | Neg | Neg | Neg | ND |
| 50 | AML | Unfavorable | Adverse | 14.47 | 9.60 | YES | No | Death | 40-60 | 30.99 | 53 | 11 | Neg | Neg | Neg | ND |
| 51 | AML | Intermediate | Good |  |  | No | No | Unkown | <40 | 1.7 | 88 | 142 | Neg | Neg | Neg | ND |
| 52 | MDS | Intermediate | Good | 54.50 | 53.67 | YES | YES | Survival | <40 | 19.34 | 72 | 18 | Neg | Neg | Neg | ND |
| 53 | MDS | Intermediate | Good | 48.53 | 47.40 | YES | No | Survival | 40-60 | 1.65 | 60 | 268 | Neg | Pos | Single | ND |
| 54 | MDS | Intermediate | Good | 46.77 | 45.40 | YES | No | Survival | <40 | 14 | 80 | 40 | Neg | Pos | Neg | Neg |
| 55 | MDS | Intermediate | Good | 34.83 |  | No | YES | Death | <40 | 2.92 | 50 | 21 | Neg | Neg | ND | ND |
| 56 | MDS | Intermediate | Good | 46.00 | 44.13 | YES | YES | Survival | 40-60 | 4 | 126 | 98 | Neg | ND | ND | ND |
| 57 | MDS | Unfavorable | Adverse | 1.23 |  | No | No | Death | >60 | 4.16 | 73 | 37 | Neg | Neg | Neg | R882 |
| 58 | MDS | Intermediate | Good | 42.53 | 41.17 | YES | No | Survival | 40-60 | 20.46 | 62 | 104 | Neg | Neg | Neg | Neg |
| 59 | MDS | Intermediate | Good |  |  | YES | No | Unkown | 40-60 | 0.61 | 71 | 78 | Neg | Neg | Neg | ND |
| 60 | MDS | Intermediate | Good | 0.67 |  | No | No | Death | <40 | 2.1 | 51 | 49 | Neg | Neg | Neg | Neg |
| 61 | MDS | Intermediate | Good | 13.53 | 8.97 | YES | No | Death | 40-60 | 1.81 | 77 | 22 | Neg | Neg | Neg | Neg |
| 62 | MDS | Intermediate | Good | 37.47 | 36.30 | YES | No | Survival | <40 | 6 | 118 | 64 | Neg | Neg | Double | Neg |
| 63 | MDS | Intermediate | Good | 37.63 | 36.23 | YES | No | Survival | 40-60 | 2.72 | 69 | 50 | Neg | Pos | Neg | R882 |
| 64 | MDS | Intermediate | Good | 23.80 | 22.73 | YES | No | Survival | 40-60 | 4.19 | 64 | 61 | Neg | Pos | Neg | Neg |
| 65 | MDS | Intermediate | Intermediate | 44.60 |  | No | No | Death | <40 | 4.14 | 106 | 50 | ND | ND | ND | ND |
| 66 | AML | Intermediate | Good | 32.63 | 31.37 | YES | No | Survival | <40 | 6.9 | 93 | 26 | Neg | Neg | Double | Neg |
| 67 | AML | Intermediate | Good | 32.60 | 31.23 | YES | No | Survival | 40-60 | 9.56 | 119 | 16 | Neg | Neg | Double | Neg |
| 68 | MDS | Intermediate | Good | 17.20 | 3.27 | YES | No | Death | >60 | 2.32 | 141 | 46 | Neg | Neg | Neg | Neg |
| 69 | MDS | Unkown | Unkown | 28.57 | 27.37 | YES | YES | Death | <40 | 22.61 | 61 | 159 | Neg | Neg | Neg | Neg |
| 70 | AML | Intermediate | Good | 5.47 |  | No | No | Death | <40 | 16.9 | 89 | 217 | Neg | Neg | Neg | Neg |
| 71 | MDS | Intermediate | Good | 30.43 | 28.87 | YES | No | Survival | >60 | 1.49 | 75 | 76 | Neg | Neg | Neg | R882 |
| 72 | MDS | Intermediate | Good | 29.70 | 28.53 | YES | No | Survival | <40 | 2.1 | 113 | 110 | Neg | Pos | Neg | Neg |
| 73 | MDS | Intermediate | Good | 29.43 | 28.23 | YES | YES | Survival | <40 | 7.7 | 87 | 166 | Pos | Neg | Neg | Neg |
| 74 | MDS | Intermediate | Good | 8.60 |  | No | No | Death | <40 | 1.78 | 46 | 70 | Neg | Neg | Neg | Neg |
| 75 | AML | Intermediate | Good | 27.60 | 26.40 | YES | No | Survival | <40 | 5.85 | 111 | 59 | ND | ND | ND | ND |
| 76 | MDS | Unfavorable | Good | 1.00 |  | No | No | Death | >60 | 1.27 | 76 | 38 | Neg | Neg | Neg | Neg |
| 77 | MDS | Intermediate | Good | 26.43 | 25.20 | YES | YES | Survival | <40 | 2.13 | 82 | 49 | Neg | Neg | Neg | Neg |
| 78 | MDS | Intermediate | Good | 26.17 | 24.20 | YES | YES | Survival | <40 | 3.39 | 72 | 480 | Neg | ND | ND | ND |
| 79 | MDS | Intermediate | Good | 25.77 | 24.37 | YES | No | Survival | <40 | 3.32 | 67 | 49 | Neg | Neg | Single | Neg |
| 80 | MDS | Intermediate | Good |  |  | No | No | Unkown | 40-60 | 0.96 | 75 | 114 | Neg | Neg | Neg | Neg |
| 81 | AML | Intermediate | Good | 4.80 | 3.30 | YES | No | Survival | <40 | 1.8 | 59 | 126 | Neg | Neg | ND | ND |
| 82 | MDS | Unfavorable | Adverse | 13.73 | 5.33 | YES | No | Death | 40-60 | 6.37 | 46 | 23 | Neg | Neg | Neg | Neg |
| 83 | MDS | Intermediate | Good |  |  | No | No | Unkown | 40-60 | 2.8 | 58 | 72 | Neg | Neg | Neg | Neg |
| 84 | MDS | Intermediate | Good | 7.57 | 5.03 | YES | YES | Death | 40-60 | 1.29 | 69 | 47 | Neg | Neg | Neg | Neg |
| 85 | AML | Intermediate | Good | 13.20 | 11.57 | YES | YES | Death | <40 | 1.31 | 70 | 65 | Neg | Pos | Neg | Neg |
| 86 | MDS | Intermediate | Good | 18.23 | 16.97 | YES | No | Survival | 40-60 | 3.5 | 71 | 93 | Neg | Neg | Neg | Neg |
| 87 | AML | Intermediate | Intermediate | 18.17 | 16.97 | YES | YES | Survival | <40 | 1.23 | 67 | 10 | ND | ND | ND | ND |
| 88 | AML | Intermediate | Good | 16.57 | 14.43 | YES | No | Survival | 40-60 | 2.59 | 77 | 133 | Neg | Neg | Neg | Neg |
| 89 | AML | Intermediate | Good | 15.13 | 12.63 | YES | No | Survival | <40 | 2.92 | 72 | 17 | ND | ND | Single | ND |
| 90 | MDS | Intermediate | Good | 10.27 |  | No | YES | Death | <40 | 3.7 | 63 | 11 | Neg | Neg | Neg | Neg |
| 91 | MDS | Intermediate | Good | 12.70 | 11.50 | YES | YES | Survival | <40 | 2.17 | 96 | 93 | Neg | Pos | Neg | ND |
| 92 | AML | Intermediate | Good | 12.57 |  | No | No | Survival | 40-60 | 1.52 | 55 | 29 | Neg | ND | Neg | ND |
| 93 | AML | Intermediate | Good |  |  | No | No | Unkown | 40-60 | 5.9 | 83 | 13 | Neg | Pos | Neg | Neg |
| 94 | MDS | Intermediate | Good | 8.67 |  | No | No | Survival | <40 | 5.5 | 85 | 36 | Neg | Neg | Neg | Neg |
| 95 | MDS | Intermediate | Good | 1.37 |  | No | No | Death | <40 | 10.85 | 71 | 152 | Neg | Neg | Neg | Neg |
| 96 | MDS | Intermediate | Good | 0.40 |  | No | No | Death | 40-60 | 9.97 | 78 | 18 | Neg | Neg | Neg | Neg |
| 97 | AML | Intermediate | Good | 7.00 | 5.50 | YES | No | Survival | >60 | 1.8 | 41 | 62 | Neg | Pos | Neg | Neg |
|  |  |  |  |  |  |  |  |  |  |  |  |  |  |  |  |  |
